# Enhancing Structural Integrity Assessment Through Non-Destructive Evaluation

**DOI:** 10.3390/ma18204748

**Published:** 2025-10-16

**Authors:** Wael Zatar, Felipe Mota Ruiz, Hien Nghiem

**Affiliations:** 1 Encova Center for Engineering and Safety, Department of Civil Engineering, Marshall University, Huntington, WV 25755, USA; nghiem@marshall.edu; 2 Department of Civil Engineering, Marshall University, Huntington, WV 25755, USA; motaruiz@marshall.edu

**Keywords:** nondestructive testing, ground-penetrating radar, reinforced concrete, reinforcement bar diameter, cover depth, box beam, bridge inspection

## Abstract

This study presents an amplitude-based non-destructive testing (NDT) approach for estimating reinforcement bar diameter in reinforced concrete members using ground-penetrating radar (GPR). The novelty of this work lies in the use of normalized amplitude-diameter-depth (NADD) relationships, which link the reflected electromagnetic wave amplitude to both rebar diameter and cover depth through an exponential attenuation model. Normalization was applied to remove the influence of varying signal energy and antenna coupling, thereby allowing consistent comparison of amplitudes across different depths and improving the reliability of amplitude-depth interpretation. The NADD equation was developed from GPR measurements obtained on a reinforced concrete slab containing bars with diameters ranging from 9.5 mm (#3 bar) to 25.4 mm (#8 bar) and then validated using data from three prestressed concrete box beams recovered from a decommissioned bridge managed by the West Virginia Department of Highways. The normalized amplitude prediction error (*E_a_*) was calculated to quantify model performance. The minimum mean error of approximately 4.7% corresponded to the 12.7 mm (#4 bar), which matched the actual reinforcement used in the beams. The results demonstrate that the proposed normalization-based approach effectively captures the amplitude-depth-diameter relationship, offering a quantitative framework for interpreting GPR data and improving the evaluation of reinforcement characteristics in existing concrete structures.

## 1. Introduction

Ensuring the strength and reliability of reinforced concrete structures is crucial across various industries, particularly as global infrastructure continues to age and experience increased loading demands. Reinforced concrete remains one of the most widely used construction materials due to its high compressive strength, durability, and versatility [1]. However, over time, factors such as material degradation, corrosion of embedded steel reinforcement, and environmental exposure can reduce its performance and safety, potentially leading to service deficiencies, structural failures, and significant economic losses.

These defects fall into two main categories: manufacturing defects and in-service defects. Manufacturing defects arise during the complex production process, which involves multiple steps where errors may occur, potentially affecting the final product’s integrity [2]. On the other hand, in-service defects develop over time as the material is exposed to operational stresses and environmental conditions during use [3]. Destructive and non-destructive testing (NDT) methods are employed to identify defects and assess the structural integrity of reinforced concrete, ensuring its reliability and service performance.

Destructive testing involves intentionally damaging or decommissioning the tested product to evaluate its properties and failure mechanisms [3]. Due to its irreversible nature, this method is typically reserved for specific applications where sacrificing a sample is necessary to gain critical insights. In contrast, NDT enables the evaluation of material integrity without causing significant damage to the structure [4].

Reinforced concrete is widely used in construction because of its versatility, cost-effectiveness, reliability, and strength. However, one of its main limitations is its low tensile strength, which is why steel reinforcement is necessary to improve its load-bearing capacity. Despite this addition, reinforced concrete structures, particularly bridges and other infrastructure subjected to harsh environmental conditions, are highly susceptible to corrosion of the reinforcement over time. Extensive corrosion can lead to severe structural issues, including delamination, spalling, reinforcement bar debonding, and a significant reduction in load capacity.

NDT techniques are widely used to evaluate the condition of materials throughout their service life, enabling the estimation of their remaining strength and longevity [5]. Due to its efficiency and reliability, NDT has become the preferred approach for assessing the condition of reinforced concrete structures [6]. Commonly employed NDT methods include visual inspection [4], ultrasonic testing [7], infrared thermography [3], and electromagnetic-based techniques [6]. These methods provide valuable information on structural integrity while preserving the functionality of the tested material.

Ground Penetrating Radar (GPR) has gained significant attention in the field of NDT. Initially, GPR was primarily used for soil exploration and mapping. However, with technological advancements and the development of higher-frequency antennas in the early 1990s, it quickly became a robust and reliable method adopted by researchers and professionals worldwide [8]. One of the earliest and most enthusiastic adopters of GPR technology was the civil engineering sector, primarily due to its widespread use in the inspection and evaluation of reinforced concrete structures [6]. GPR uses electromagnetic (EM) pulses to scan beneath surfaces. Reflected signals are captured and processed into images that reveal internal structures [9]. Compared to conventional electromagnetic methods, GPR offers deeper penetration, can detect multiple reinforcement layers, and can identify non-metallic reinforcement, making it a more versatile tool for evaluating complex concrete structures.

Due to its capabilities in assessing reinforced concrete structures, GPR has undergone extensive evaluation in the civil engineering field regarding its reliability and efficiency for structural assessments. Some of the key applications of GPR include assessment of bridge decks [10], mapping of steel reinforcement bar layout within reinforced concrete structures [11,12,13], estimation of internal feature depths and concrete cover [14,15], monitoring cracks [16], visualization of internal deficiencies [17], detection and estimation of reinforcement bar corrosion extent in concrete slabs [18,19], assessment of anomalies in concrete structures with fiber reinforced polymer-wrap fiber-reinforcement [20,21,22]. These applications demonstrate the diverse range of areas where GPR has proven effective.

Although GPR and other NDT techniques are effective at detecting reinforcement steel and internal defects, assessing the condition of reinforcement bars, especially determining their diameter, still largely depends on destructive testing methods. Non-destructive approaches for accurately estimating reinforcement bar diameter are still in the early stages of development. This highlights the continuing need for advancements in non-destructive evaluation techniques for reinforced concrete structures.

Previous research has estimated reinforcement bar diameter by analyzing electromagnetic (EM) hyperbolic reflections using a template selection algorithm [23], which matches these reflected hyperbolic shapes with biquadratic equations [24]. However, these methods were tested under ideal conditions, specifically using concrete slabs with simple reinforcement layouts. When applied to more complex structures, such as prestressed concrete box beams, significant limitations were observed [25]. These examples highlight the challenges of implementing these techniques in real-world scenarios and underscore the need for further advancements in NDT methods for assessing reinforced concrete.

Methods to determine the velocity of EM waves traveling through various materials are discussed in the literature [26,27]. However, these methods require prior knowledge of the bar diameter in reinforced concrete structures. Since this study is focused on scenarios where the bar diameter is unknown, the conventional techniques described in the literature are not suitable. To address this limitation, the velocity of the electromagnetic wave can be calculated using the reflections from the bottom face of the concrete specimen. If the reflected signal is weak and the boundaries of the concrete slab cannot be accurately determined, placing a metal plate on the bottom face of the material can enhance the EM reflection [23].

This study presents a methodology for estimating reinforcement bar diameter in concrete using a normalized amplitude-diameter-depth (NADD) analysis of GPR signals. In this approach, the raw reflected amplitudes are normalized with respect to the maximum amplitude within each scan to reduce the influence of antenna coupling, surface condition, and energy variation, thereby allowing amplitude comparisons across different depths and specimens. This normalization enhances prediction consistency by emphasizing the relative attenuation behavior, which is primarily governed by material properties and reinforcement bar geometry rather than measurement artifacts [28]. By establishing empirical relationships between EM reflection amplitudes, cover depth, and diameter, standardized reference curves and equations were developed. GPR data from slab specimens were analyzed using a custom Delphi-based algorithm to automate amplitude extraction and depth estimation. The approach was subsequently validated through field applications on prestressed concrete box beams from a decommissioned bridge in West Virginia. Results demonstrated that the predicted reinforcement bar diameters matched the actual reinforcement details obtained from the structural drawings, confirming the method’s validity for non-destructive structural assessment.

## 2. Research Significance

Accurate identification of reinforcement bar diameter is essential for evaluating mechanical resistance and safety of in-service reinforced concrete components. However, current NDT methods often struggle to provide precise estimations of bar diameter, especially in complex or aged structures [24,25,26,27,28]. These limitations hinder engineers from making fully informed decisions about structural rehabilitation or replacement.

This study introduces a GPR-based methodology for estimating reinforcement bar diameter through normalized amplitude-diameter-depth (NADD) analysis. The innovation lies in the application of tailored NADD curves, developed from an exponential attenuation model, to correlate normalized reflection amplitudes with bar diameter and depth. The normalization process minimizes the influence of antenna coupling, surface roughness, and local material variations, enabling consistent amplitude comparison across different depths and specimens. As a result, the proposed approach provides improved prediction consistency and robustness compared with analyses based solely on unnormalized amplitude data. The method offers a quantitative, model-driven enhancement to traditional amplitude-based GPR interpretation, supporting more reliable assessment of reinforcement characteristics in concrete structures.

Despite the promising performance of the proposed methodology, several limitations must be acknowledged. The analysis is currently calibrated for conventional reinforced concrete members and may not directly apply to structures with significant variations in material composition, such as steel fiber reinforced concrete (SFRC), due to potential differences in electromagnetic response. Additionally, environmental and construction variabilities, such as moisture content, corrosion, or embedded material heterogeneity, could affect amplitude-based estimations. These factors will be the focus of future work aimed at improving the generalizability and robustness of the method in diverse field conditions.

## 3. Signal Processing and Analytical Methodology

### 3.1. GPR System Architecture and Signal Characterization

GPR is a subsurface imaging technology that consists of both transmitting and receiving antennas. The transmitting antenna emits high-frequency EM pulses into materials such as soil, concrete, or rock. As these EM waves travel through the medium, they encounter interfaces between materials with different EM properties, such as variations in composition, moisture content, or the presence of embedded objects, which cause the waves to reflect. The receiving antenna captures these reflected signals and sends them to a computer system. This system processes and interprets the reflected EM pulses to generate detailed two-dimensional images that reveal the internal structure of the material. This technique allows users to detect and visualize subsurface anomalies or objects without the need for excavation or physical intrusion [9].

The frequency of the wave varies based on its application. Lower frequencies can penetrate deeper beneath the surface, but they result in a lower signal-to-noise ratio and reduced image resolution. On the other hand, higher frequencies yield more detailed images, but only at shallower depths. For most civil engineering applications, GPR operates within a frequency range of 20 MHz to 2.5 GHz [9,29,30]. The propagation of EM waves is significantly affected by two key physical properties: the dielectric constant and the electrical conductivity of the material.

The dielectric constant plays a crucial role in determining the speed at which EM pulses travel through different media, which is essential for GPR data analysis. In air, EM waves move at a speed close to that of light, resulting in a dielectric constant of 1. In contrast, when traveling through water, EM waves move at approximately one-ninth the speed of light, giving water a dielectric constant of 81.

For most construction materials, including reinforced concrete, the wave velocities typically range from 88.9 to 177.8 mm per nanosecond, translating to dielectric constants of 12 and 3, respectively. By calculating the velocity of the EM waves, the depth of internal features can be estimated by multiplying the wave velocity by the two-way travel time of the pulses from the transmitter to the receiver.

Alongside the material’s dielectric constant, the conductivity of the material plays a vital role in EM wave propagation. Low-conductivity materials, such as dry sand, dry concrete, and ice, are great examples of materials that allow for greater penetration with minimal energy loss. Conversely, high-conductive materials, such as salt water and wet concrete, absorb EM energy before the pulses can travel far through the medium. These characteristics make materials such as wood, concrete, and asphalt excellent candidates for GPR evaluation, contributing to the widespread adoption of this NDT in civil engineering applications.

**Figure 1:**
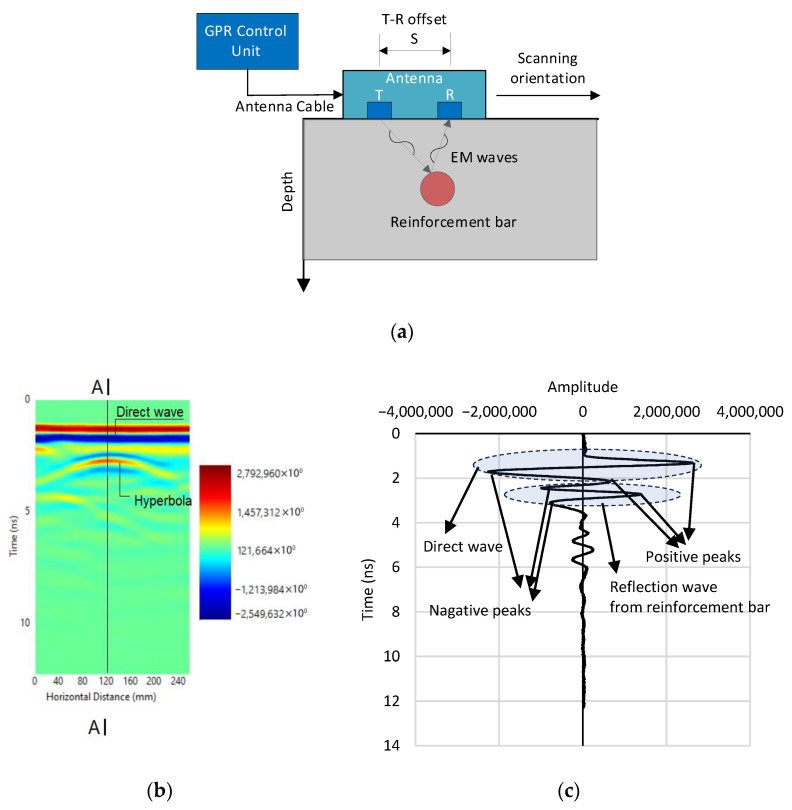
GPR System Architecture and Signal Characterization: (**a**) A basic principle of ground-penetrating radar technique; (**b**) Radargram of GPR data; (**c**) One-dimensional time-domain signal (Section A-A).

Figure 1a illustrates the fundamental operating principle of a ground-penetrating radar (GPR) system, which typically consists of three main components: a control unit, a transmitter, and a receiver. The control unit governs the system’s operation and data acquisition. The transmitter emits electromagnetic (EM) waves into the subsurface, while the receiver captures the reflected signals. As the EM waves propagate through a material, they maintain a velocity determined by the material’s dielectric constant. When these waves encounter a material with a different dielectric constant, part of the energy is reflected to the receiver. These reflections occur at interfaces such as air voids, embedded reinforcement bars, water pockets, or pipes. The greater the difference in dielectric constants between the two materials, the stronger the reflected signal. For example, a reinforcement bar embedded in concrete generates a pronounced reflection due to the significant contrast in both dielectric constant and electrical conductivity between steel and concrete, as shown in Figure 1a.

GPR data are typically displayed as a two-dimensional image called a radargram, which maps the antenna’s horizontal scanning position against the signal travel time or depth. The horizontal axis represents the scanning distance, while the vertical axis corresponds to the travel time or depth of internal reflections. The amplitude of reflected signals is visualized using a color scale, as illustrated in Figure 1b. In this scale, dark blue represents the strongest negative amplitude, while dark red indicates the strongest positive amplitude. The two horizontal red-blue bands at the top of the radargram indicate the direct wave traveling along the antenna-material interface, while the characteristic hyperbolic reflection in red signifies a subsurface feature such as a reinforcement bar.

Figure 1c presents a signal amplitude array extracted from the vertical cross-section A-A of the radargram shown in Figure 1b. This array is displayed as a one-dimensional time-domain signal, where the vertical axis represents the signal travel time and the horizontal axis represents the amplitude of the EM wave. This representation provides a view of the signal strength and waveform characteristics at a specific scan location, facilitating further quantitative analysis of reflected features.

This study utilized the GSSI SIR-4000 ground-penetrating radar system, a high-performance control unit designed for advanced data acquisition in non-destructive testing applications. The system was paired with a 1.6 GHz high-frequency antenna, suitable for high-resolution imaging of near-surface features such as reinforcement bars in concrete. The antenna had a transmitter–receiver (T–R) offset (S) of 58 mm, facilitating accurate depth and amplitude detection. The SIR-4000’s robust signal processing capabilities ensured reliable collection and interpretation of reflection amplitudes across varying concrete conditions [9].

### 3.2. Analytical Methodology for Reinforcement Bar Localization

The shape of the hyperbola is a critical factor in GPR data processing. This shape is influenced by two main parameters: (1) scan spacing, where shorter scan spacings (meter per scan) result in wider parabolas, and (2) wave velocity, where lower dielectric constants (indicating higher EM wave velocity) lead to wider parabolas, and vice versa. Moreover, larger targets produce brighter reflections; however, the change in the hyperbola shape for different target sizes is minimal for any diameter under 50.8 mm, as these target sizes are a fraction of the EM wavelength. The theoretical equations governing the hyperbola are derived based on the following assumptions:

- The positive peaks of the reflected waves from the reinforcement bar correspond to the negative peaks of the transmitted wave, indicating a phase reversal.
- The transmitted waves reflect at the surface of the reinforcement bar along the shortest two-way travel path.

**Figure 2:**
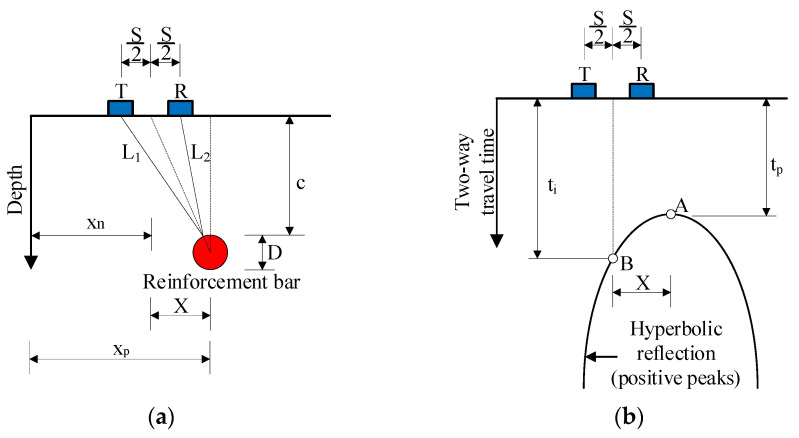
Reflected EM wave from a reinforcement bar: (**a**) EM wave travel path; (**b**) hyperbolic reflection.

The length of the traveling path of the signal reflected from a reinforcement bar can be obtained in the following equations, as shown in Figure 2a:

(1a) $$L_{1}=\sqrt{c^{2}+{\left(X-\frac{S}{2}\right)}^{2}}\;$$

(1b) $$L_{2}=\sqrt{c^{2}+{\left(X+\frac{S}{2}\right)}^{2}}\;$$

where X = x_p_ − x_n_ is the distance in the horizontal direction between the reinforcement bar and the center line of the transmitter and receiver; c is the cover depth of the reinforcement bar; S is the spacing of the transmitter and receiver (T-R offset); x_p_ is the horizontal coordinate of the reinforcement bar center, x_p_ = (n_p_ − 1)S_s_; x_n_ is horizontal coordinate of the antenna, x_n_ = (n − 1)S_s_; where n_p_ and n are scan number at reinforcement bar center and the center line of the transmitter and receiver, respectively; S_s_ is scan spacing in scanning orientation.

Considering the peak point of the hyperbola, the two-wave travel time is expressed by Equation (2) as:

(2) $$t_{p}-t_{0}=\frac{\sqrt{4c^{2}+S^{2}}}{V_{s}}$$

where t_0_ is time zero and t_p_ is the peak time of the hyperbola. Consider a point on the hyperbola (t_i_), the two-wave travel time can be obtained by employing Equation (4):

(3) $$t_{i}-t_{0}=\frac{L_{1}+L_{2}}{V_{s}}$$

Substituting Equations (1) and (3) into Equation (4) results in producing Equation (4):

(4) $$t_{i}=\left(t_{p}-t_{0}\right)\frac{\sqrt{c^{2}+{\left(X-\frac{S}{2}\right)}^{2}}+\sqrt{c^{2}+{\left(X+\frac{S}{2}\right)}^{2}}}{\sqrt{{4c}^{2}+S^{2}}}+t_{0}$$

Equation (4) represents the theoretical hyperbolic reflection generated by a reinforcing bar embedded within concrete, as illustrated in Figure 2b. The general shape of the hyperbola primarily depends on the wave velocity and the cover depth, while the effect of bar diameter on the curvature is relatively small compared with these dominant factors. Consequently, variations in hyperbolic geometry among different bar sizes are minor and are typically less influential than amplitude characteristics in distinguishing reinforcement diameters.

**Figure 3:**
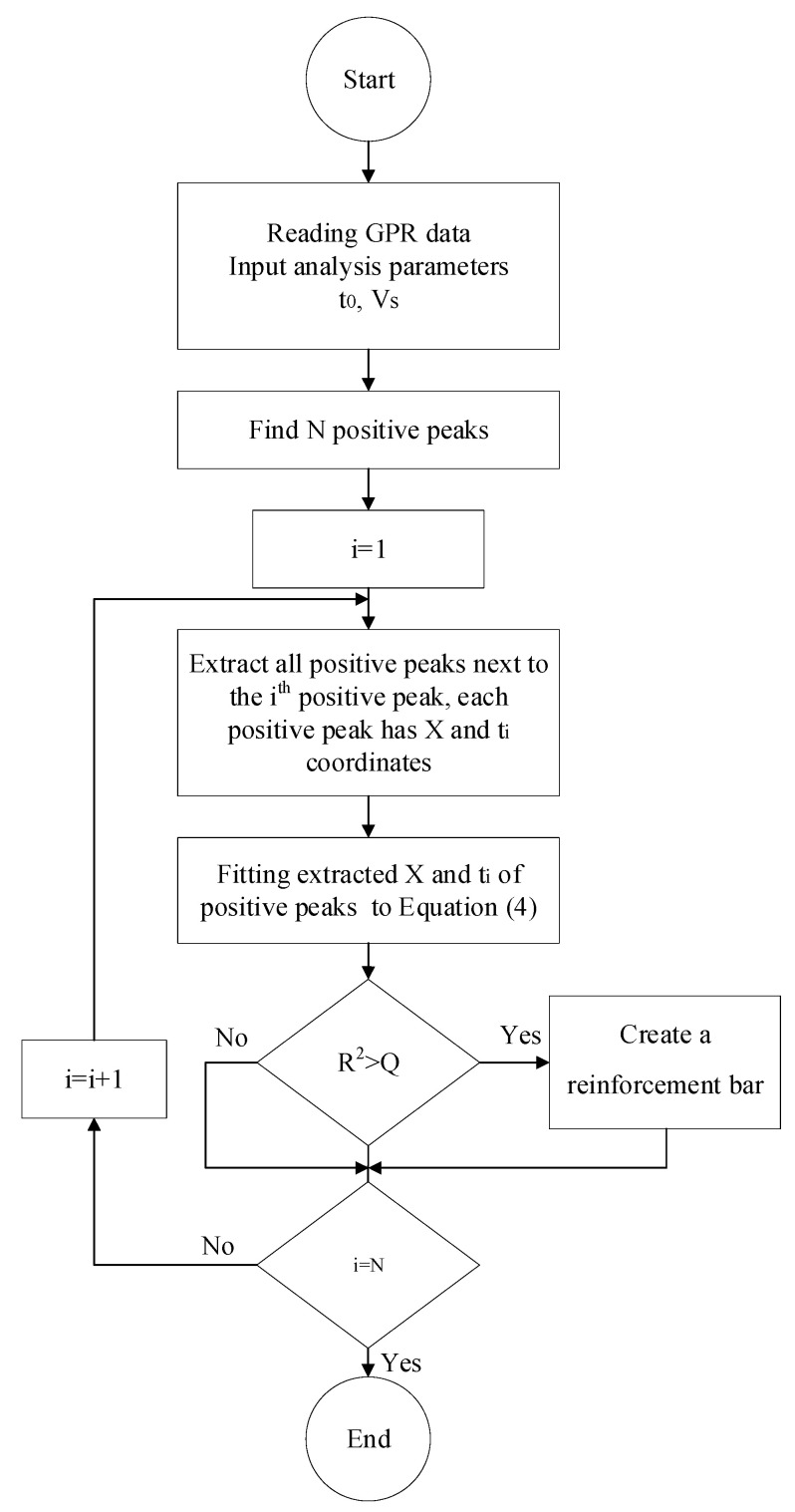
Flowchart of the automated reinforcement bar detection algorithm from GPR data based on Equation (4) fitting and R^2^ threshold.

A flowchart of the algorithm used to identify reinforcement bars is presented in Figure 3. The algorithm begins by reading the GPR data along with user-defined parameters such as time-zero and wave velocity. It then iteratively locates positive amplitude peaks and extracts adjacent peaks that may belong to the same hyperbolic reflection. The spatial (*X*) and temporal (*t_i_*) coordinates of these peaks are fitted to Equation (4). The goodness of fit is evaluated using the coefficient of determination (*R*^2^), and if it exceeds a predefined threshold *Q*, a reinforcement bar is registered at the vertex of the fitted hyperbola, and the corresponding positive amplitude at that location is also recorded. This positive amplitude (hyperbola vertex amplitude) is subsequently used in the next section to estimate the reinforcement bar diameter based on the best-fit normalized amplitude-diameter-depth (NADD) curve.

An automatic algorithm for reinforcement bar picking was developed for this study. This algorithm enables the automatic identification of EM amplitude peaks that correspond to the locations of reinforcement bars, as well as the calculation of reinforcement bar depth and concrete cover. It also generates tables that relate EM wave amplitude to depth reflected from reinforcement bars.

Following the reinforcement bar identification workflow (Figure 3), reliable depth estimation requires determining two critical parameters: the time-zero and the wave velocity (*V_s_*) in concrete. In this study, time-zero was determined following the approach of Zatar et al. [24], which identifies the zero-time point from the negative peak of the direct wave rather than requiring a metal plate for calibration. This method aligns the onset of the transmitted and reflected signals by accounting for the signal’s phase reversal upon reflection, allowing the time zero to be computed from the travel time of the direct wave in air. To calculate *V_s_*, reflections from the bottom boundary of the concrete specimen were used. When reflections from the opposite surface are too weak for reliable detection, a metal plate is placed beneath the slab to enhance the EM wave reflection [23], thereby improving the accuracy of velocity estimation. These values of *t*_0_ and *V_s_* are input into the algorithm described in Figure 3 for further processing.

### 3.3. Reinforcement Bar Diameter Estimation Using GPR NADD Relationship

This section outlines the methodology used to estimate the diameter of reinforcement bars based on GPR signal characteristics. The specific form of the amplitude-depth equation is derived empirically through curve fitting. The hyperbola vertex amplitude, illustrated in Figure 2b, defined as the positive signal amplitude corresponding to the apex of the hyperbolic reflection identified in Section 3.2, is primarily influenced by the bar diameter and attenuates with increasing depth. To mitigate depth-related effects, this amplitude is normalized by the amplitude of the direct wave, resulting in a dimensionless ratio that reduces attenuation bias. The normalized amplitude values are then plotted against the corresponding cover depths to produce NADD curves, which serve as the basis for estimating reinforcement bar diameters.

**Figure 4:**
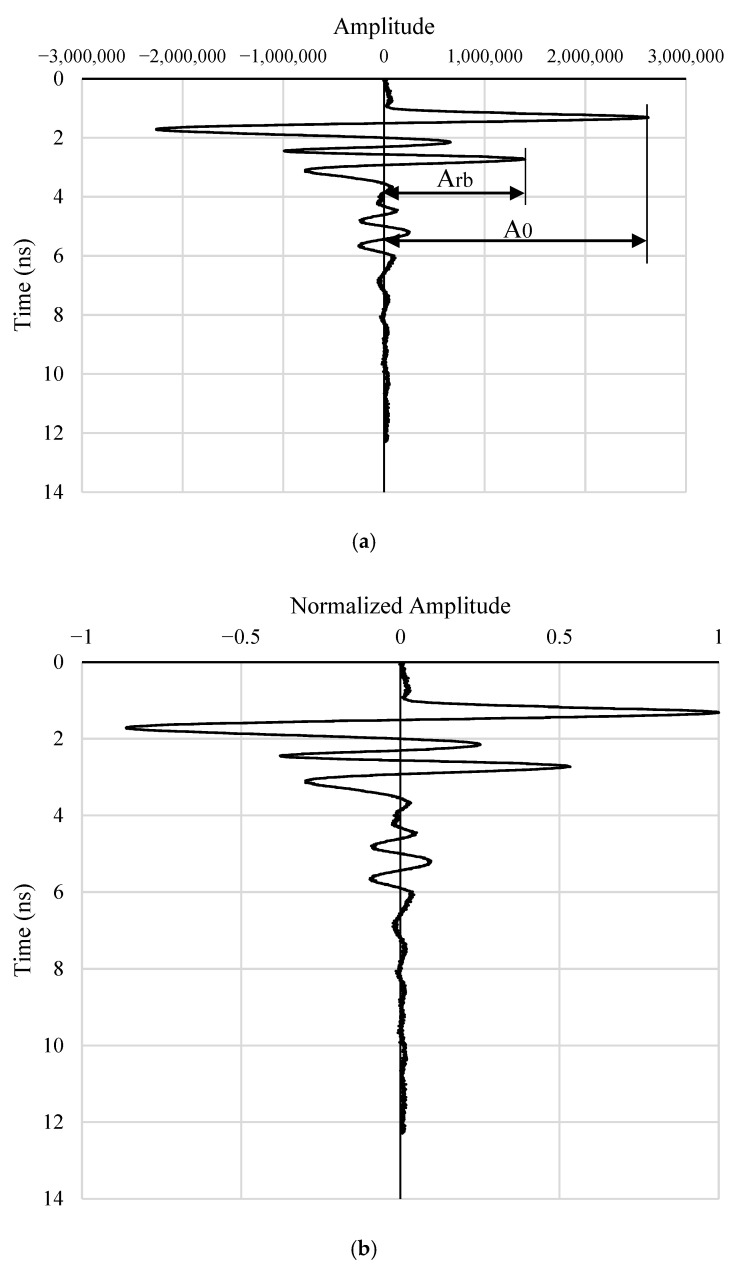
Definition of normalized amplitude in a signal amplitude array: (**a**) Raw amplitude profile showing the reflection from the reinforcement bar; (**b**) Corresponding normalized amplitude used to construct the amplitude–diameter–depth (NADD) relationships.

The signal amplitude array shown in Figure 4a is obtained from a scan where the transmitter-receiver centerline is aligned directly above the reinforcement bar. This array includes both the direct wave amplitude (*A*_0_) and the hyperbola vertex amplitude (*A_rb_*), which corresponds to the peak of the reflected signal from the reinforcement bar. To account for signal attenuation with depth and isolate the effect of bar diameter, the amplitude *A_rb_* is normalized by the direct wave amplitude *A*_0_, yielding a dimensionless normalized amplitude *A*, defined as $A=A_{rb}/A_{0}$, as illustrated in Figure 4b.

The equation incorporating *α*(*D*) and *A*_1_(*D*) is used to model how the reflected GPR signal amplitude decays with depth while accounting for the influence of reinforcement bar diameter. In ground-penetrating radar (GPR), the amplitude of the reflected wave from a reinforcement bar decreases exponentially as the wave travels through concrete due to attenuation, which arises from material absorption, scattering, and reflection losses. However, experimental observations show that the attenuation rate and initial amplitude are not constant, they vary with the diameter of the reinforcement bar. Larger reinforcement bars typically produce stronger reflections (higher *A*_1_) but may also experience different attenuation characteristics (*α*) because of their geometry and reflection cross-section. Therefore, the following equation is used to capture these relationships:

(5) $$A=A_{1}(D)e^{-\alpha (D)c}$$

where *α*(*D*) denotes the amplitude attenuation rate per millimeter, and *A*_1_(*D*) represents the normalized amplitude at zero depth (intrinsic reflection strength), both dependent on the reinforcement bar diameter. Equation (5) thus forms the basis of the NADD equation, used to estimate reinforcement bar diameter from GPR data. To determine *α*(*D*) and *A*_1_(*D*) from test results, the natural logarithm was applied to both sides of Equation (5), resulting in:

(6) $$lnA=ln\left[A_{1}\left(D\right)\right]-\alpha (D)c$$

Equation (6) was thus obtained, representing a linear relationship between the measured amplitude and depth. The slope of the line corresponding to *α*(*D*) while the intercept represents *ln*[*A*_1_(*D*)].

## 4. Experimental Program

### 4.1. Concrete Slab Specimen

**Figure 5:**
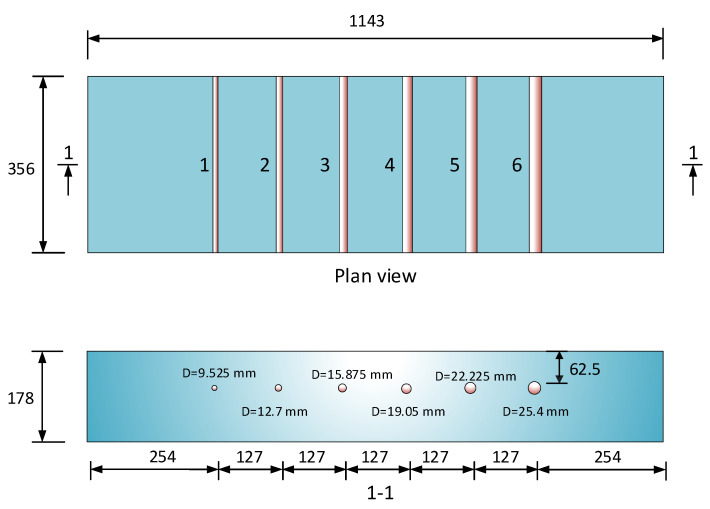
Concrete slab specimen.

This study examined a reinforced concrete slab containing six reinforcement bars (as numbered from 1 to 6 in Figure 5) with diameters of 9.5 mm, 12.7 mm, 15.9 mm, 19.1 mm, 22.2 mm, and 25.4 mm, all embedded at a uniform depth of 62.5 mm as specified in the design. The spacing between the reinforcement bars was 127 mm. Figure 5 provides a detailed overview of the specimen configuration.

All reinforcement bars used in the slab were visually inspected prior to casting and showed no evidence of corrosion, such as surface pitting, rust, or scaling. Although no mass-loss tests were performed, the bars were assumed to be uncorroded based on their physical condition and brief storage in a controlled environment. The slab was cast using ready-mix concrete supplied by a local commercial producer to ensure consistency and quality control. The concrete had a specified 28-day compressive strength of 35 MPa, typical of structural concrete used in reinforced members. Because only a single slab was examined, the proposed method’s external applicability to structures with varying concrete compositions may be limited. Future experiments should include concrete mixes with different water-cement ratios, aggregate types, and moisture contents to evaluate the robustness of the approach under diverse field conditions.

### 4.2. Data Collection

The concrete slab specimen underwent a thorough examination. GPR scans were performed along a survey grid comprising three longitudinal lines, spaced 305 mm apart, which intersected the reinforcement bars perpendicularly. The GPR-exposed surface of the slab was analyzed to collect data supporting the study’s objectives. Table 1 detail the parameters for the GPR device in collecting data for the concrete slab specimens.

### 4.3. Best-Fit Functions

**Figure 6:**
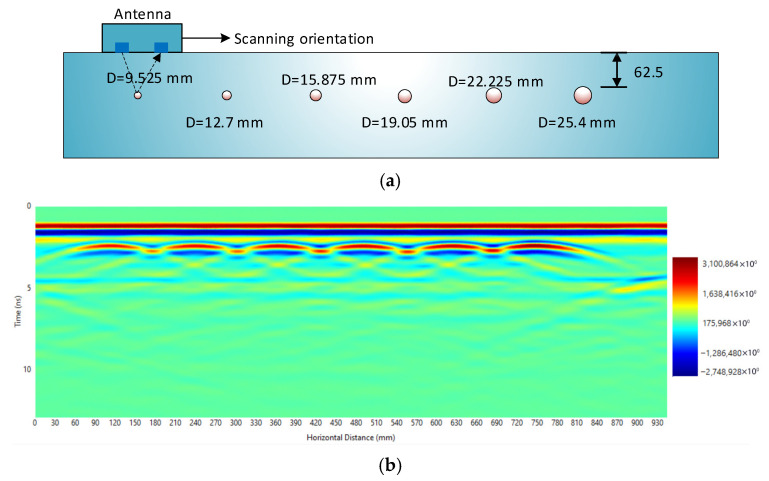
GPR scanning of slab: (**a**) Antenna location and scanning orientation; (**b**) Radargram.

To establish the amplitude-diameter-depth function in Equation (5), fitting approaches were applied using data from specimen, which incorporated multiple reinforcement bar diameters under consistent testing conditions. The GPR survey was conducted from the exposed surface, where all reinforcement bars were embedded at approximately the same depth, as shown in Figure 6. The monitoring pathway and corresponding radargram, where the color intensity column represents the reflected signal amplitudes are also presented in Figure 6, with a color scale column indicating the signal amplitudes.

**Figure 7:**
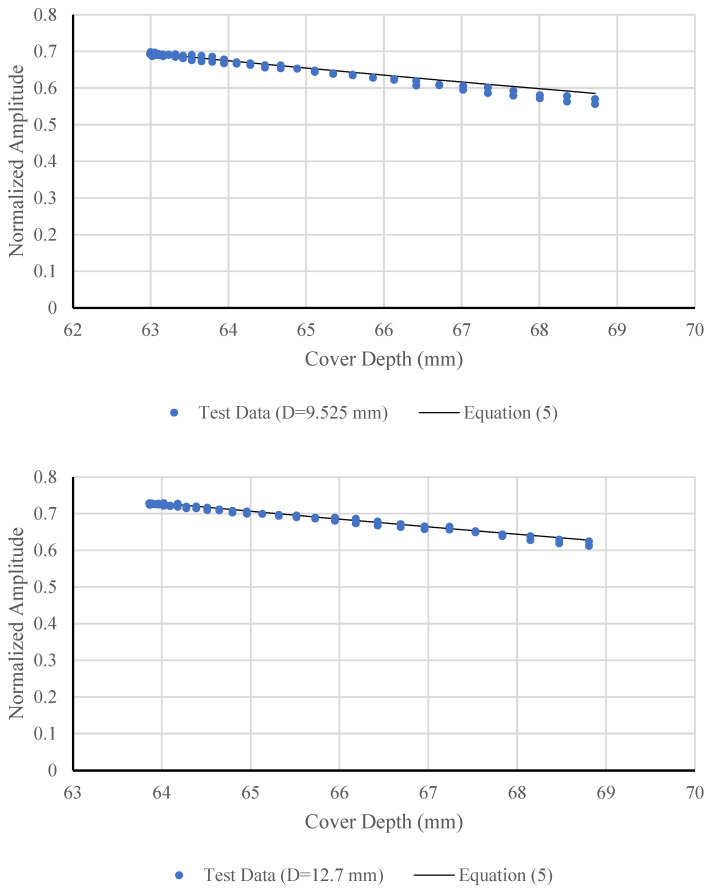

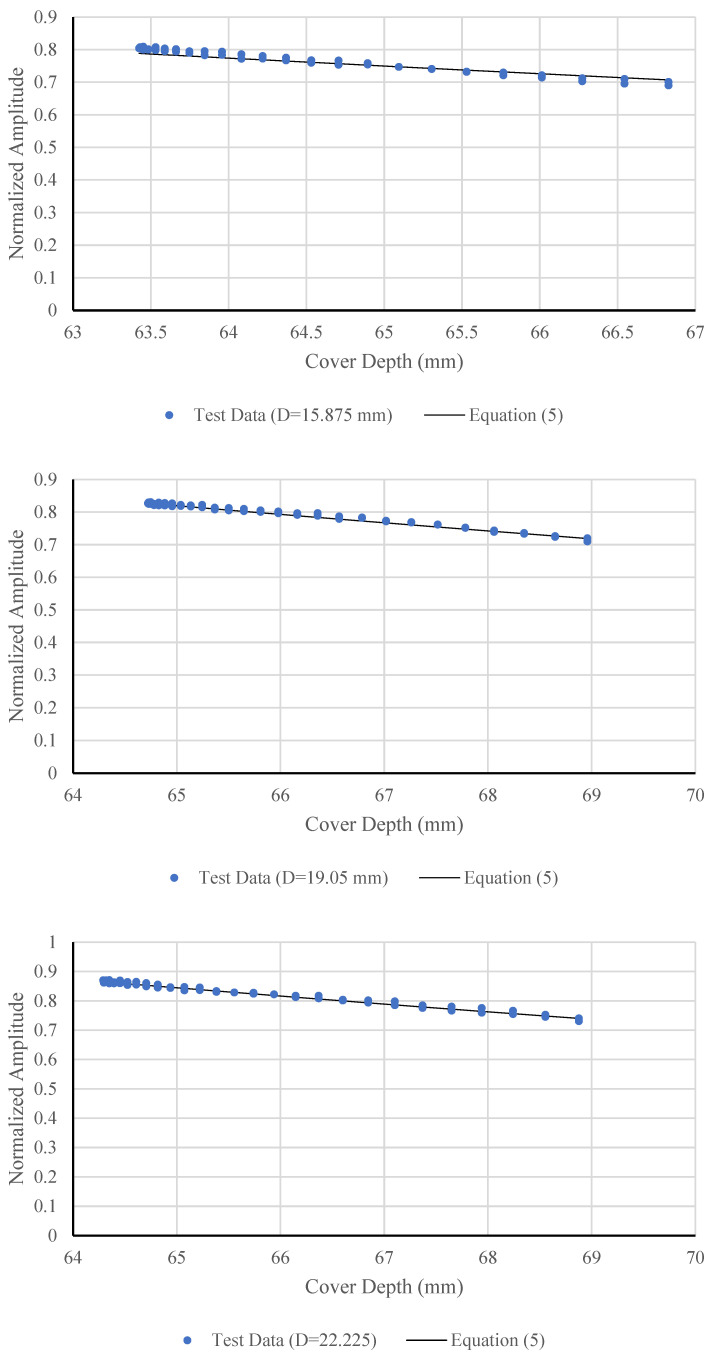

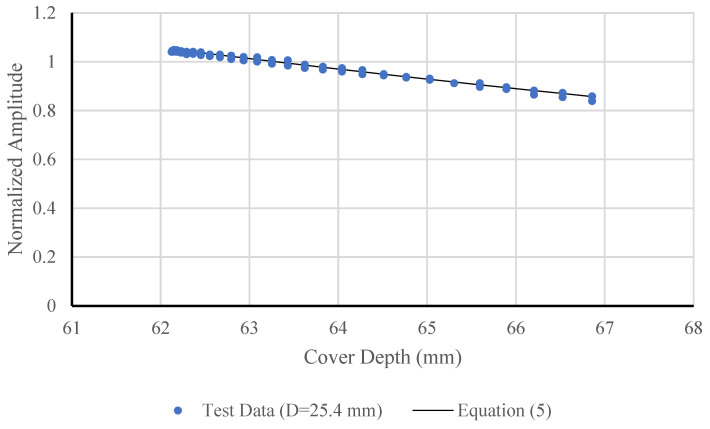
Normalized amplitudes and best fit lines.

As the antenna moves across the concrete slab surface, the distance between the antenna and each reinforcement bar changes, causing corresponding variations in the amplitude of the reflected signal. This variation establishes the amplitude-depth relationship. To minimize interference from adjacent reinforcement bars, the distance range considered in the analysis was restricted to the region producing a clearly linear portion of the amplitude-depth relationship. The normalized amplitude-depth responses, which are strongly influenced by reinforcement bar diameter, were extracted and are shown in Figure 7 (using $t_{0}=1.3\;\mathrm{ns}\;$ and $V_{s}=125\;\mathrm{mm}/\mathrm{ns}$). Equation (5) was also plotted in Figure 7 for reinforcement bars with diameters ranging from 9.525 mm to 25.4 mm, and the corresponding values of *α*(*D*), *A*_1_(*D)*, and R^2^ are summarized in Table 2.

As shown in Table 2, both *α*(*D*) and *A*_1_(*D*) increase almost linearly with bar diameter from 9.525 mm to 22.225 mm, indicating a consistent relationship between bar size and amplitude attenuation behavior. The corresponding R^2^ values (0.983–0.987) demonstrate a strong linear correlation between the experimental data and the fitted exponential model across this diameter range. A sharp increase occurs at 25.4 mm, likely due to multiple reflections or near-field effects associated with the large bar size. This observation suggests that the amplitude-diameter relationship remains approximately linear only within the mid-diameter range, while larger diameters may require nonlinear calibration to achieve more reliable amplitude-based diameter prediction in field applications.

## 5. Field Application and Validation on Bridge Beams

**Figure 8:**
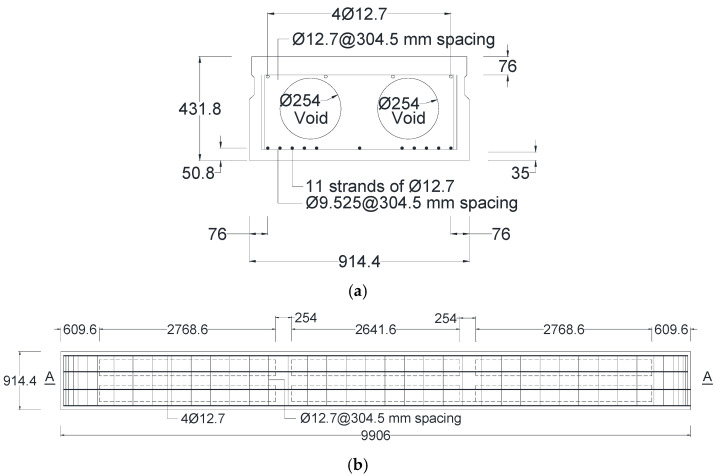

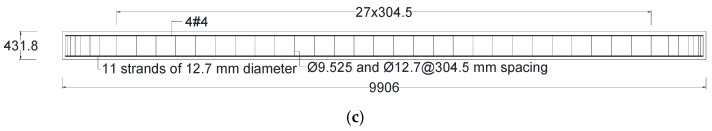
Prestressed concrete box beams: (**a**) Cross-section; (**b**) Plan view; (**c**) Section A-A.

The final step of this study involved applying the developed equations to calculate the normalized amplitude reflections from various sizes of reinforcement bars in precast prestressed concrete box beams. The West Virginia Division of Highways (WVDOH) provided three precast, prestressed concrete box beams measuring 9.906 m in length, 0.91 m in width, and 0.432 m in height, as shown in Figure 8. These beams were salvaged from a decommissioned bridge in Cabell County, West Virginia. Each beam was reinforced with four longitudinal bars at the top with a diameter of 12.7 mm, stirrups spaced at 304.5 mm intervals, two groups of five prestressed strands at the bottom, and a centrally positioned prestressed strand. Two 254 mm diameter air voids were incorporated, each divided into three segments to reduce self-weight, as shown in Figure 8a. Transverse holes located between the segments accommodated a tendon used to tie all three beams together during service. The plan view and vertical section A-A in Figure 8b,c show the detailing of the main reinforcement bar, strands, and stirrups.

The precast prestressed concrete box beams analyzed underwent a thorough examination. For this study, only the scans of the exposed surface were analyzed, due to the closely spaced prestressed strands, which require further investigation to determine their diameter with higher precision. The exposed surface scans provided critical data regarding the dimensions of the top reinforcement bars and the stirrups that were designed for shear reinforcement. For amplitude-based diameter estimation, GPR scans were primarily performed in the longitudinal direction, perpendicular to the stirrups. This orientation was chosen to maximize hyperbolic clarity and ensure consistent comparisons across specimens. While longitudinal reinforcement bars were present, their orientation and reflections were not analyzed.

**Figure 9:**
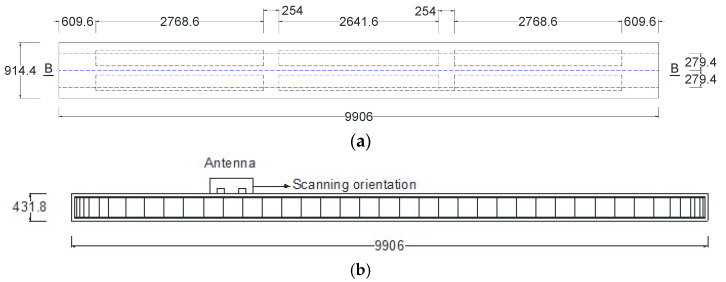
Prestressed concrete box beam survey grid: (**a**) Exposed face longitudinal (blue) scanning lines; (**b**) Section B-B.

For each beam, three parallel GPR scan lines were conducted: one along the centerline and two additional lines spaced 279.4 mm to either side of the centerline, as illustrated in Figure 9a. The antenna was positioned on the exposed surface of the beam and moved longitudinally to capture reflections from the internal stirrups, as shown in Figure 9b. The technical specifications and configuration parameters of the GPR system used in this study are provided in Table 1 and Table 3.

**Figure 10:**
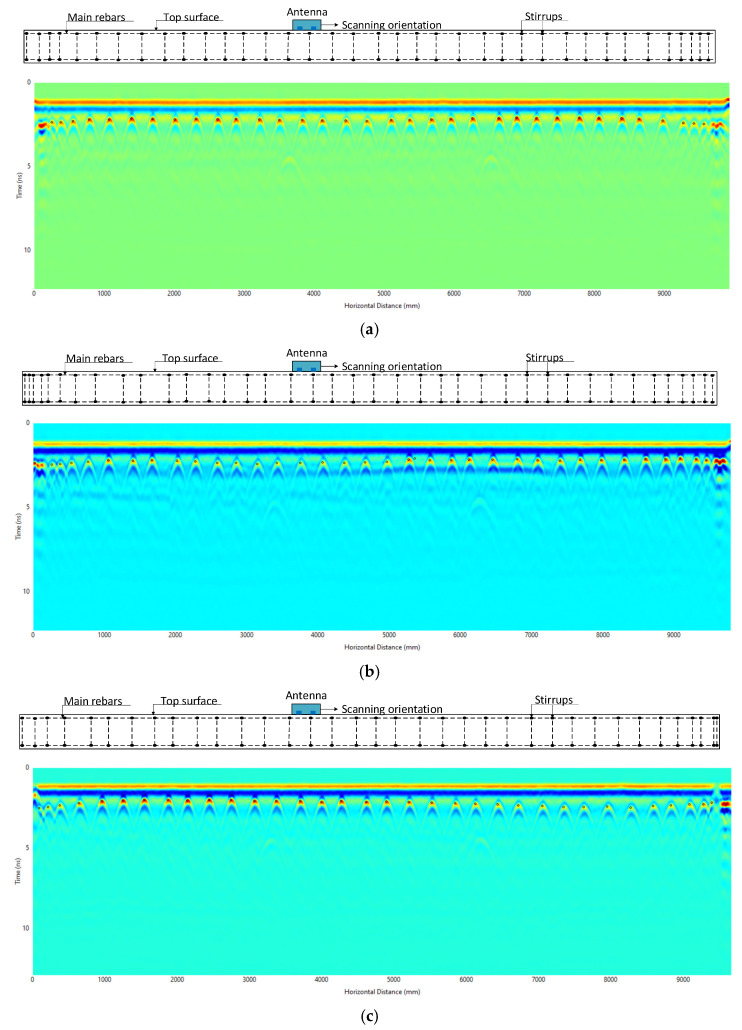
Radargram of the beams from exposed surface scanning along the longitudinal direction: (**a**) Radargram for Beam #1; (**b**) Radargram for Beam #2; (**c**) Radargram for Beam #3.

The analyses were conducted on all three beams to extract the normalized amplitude and cover depth of the reinforcement bars. The input parameters used for the analysis are summarized in Table 4 where the time zero and wave velocity determined using methods described in Section 3.2. The corresponding radargrams for each beam are shown in Figure 10, where the locations of the reinforcement bars are marked with black dots. At these marked locations, the normalized amplitudes were extracted. The resulting data will be used to compare against the established NADD curves for validation purposes.

**Figure 11:**
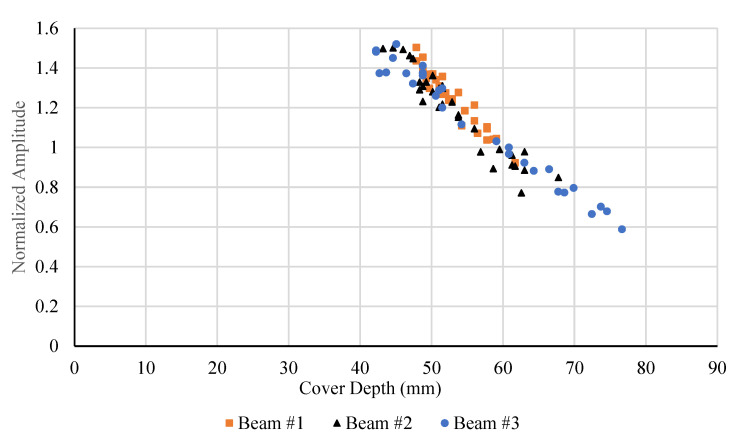
Normalized amplitudes from test data for all beams.

Figure 11 presents the variation in normalized amplitudes with cover depth for Beams #1, #2, and #3. Each data point in Figure 11 represents a normalized amplitude measurement at a specific bar location within the beam. Stirrup bars were selected as the primary monitoring targets. Despite minor scatter, all three data sets exhibit a consistent exponential decay trend, indicating that the normalized amplitude decreases systematically with increasing cover depth. This behavior reflects the expected attenuation of the GPR signal as the travel path through concrete increases.

Beams #1 and #2 show nearly overlapping trends, suggesting consistent material properties and uniform reinforcement placement. Beam #3 displays slightly lower amplitudes at equivalent depths, which may be attributed to localized variations in concrete density, surface roughness, or coupling efficiency between the antenna and the concrete surface. The strong correlation among the three beams confirms the repeatability of the amplitude–depth relationship and validates the use of the proposed attenuation model for predicting signal reduction with increasing cover.

Overall, the results demonstrate that normalized amplitude provides a reliable indicator of cover depth, with good agreement among different specimens. The consistent exponential decay trend also supports the formulation of a unified attenuation model applicable across multiple beams under similar material and testing conditions.

To identify the reinforcement bar diameter from GPR measurements, a comparative approach was employed using the calibrated amplitude–depth relationship expressed in Equation (5). For each candidate diameter D, the predicted amplitude at a given cover depth c was computed from:

(7) $$A_{p}(D,c)=A_{1}(D)e^{-\alpha (D)c}$$

where *A*_1_(*D*) and *α*(*D*) (mm^−1^) were obtained experimentally from the slab. The predicted amplitudes were then normalized and compared with the measured normalized amplitudes *A_m_* from beam tests. The difference between predicted and measured values was quantified by the normalized amplitude prediction error,

(8) $$E_{a}=\frac{\left|A_{m}{-A}_{p}(D,c)\right|}{A_{m}}\times 100\%$$

For each reinforcement location, *E_a_* was evaluated for all possible diameters, and the diameter corresponding to the minimum mean error across all cover depths was identified as the most probable bar diameter. This approach allows diameter estimation based solely on the amplitude-depth attenuation characteristics, ensuring that the comparison accounts for both signal attenuation and reflection strength. The method also enables quantitative assessment of prediction reliability by analyzing the distribution of *E_a_* values across different diameters and cover ranges.

**Figure 12:**
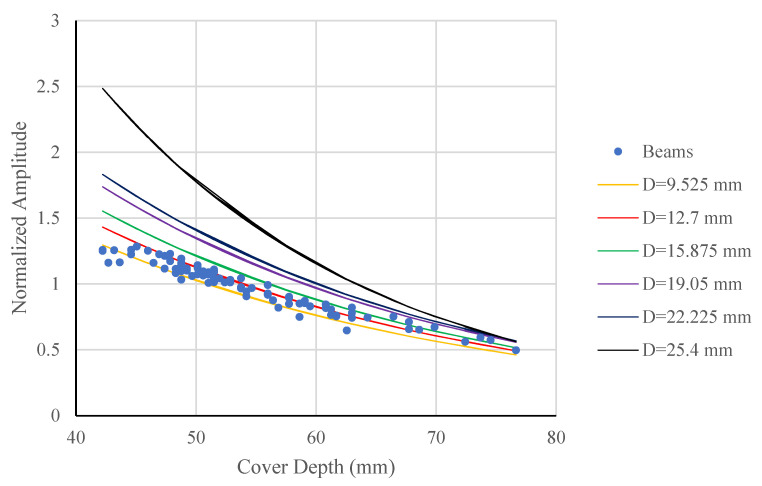
Comparison of test results and the proposed equation for all beams.

Figure 12 compares the measured normalized amplitudes from the beams with the predicted attenuation curves for reinforcement diameters ranging from 9.525 mm to 25.4 mm. All curves exhibit an exponential decrease in amplitude with increasing cover depth, consistent with the theoretical attenuation behavior of GPR signals in concrete. Among the curves, the line corresponding to 12.7 mm (red) aligns most closely with the experimental points over the entire depth range, confirming that this diameter provides the best representation of the measured amplitude-depth trend.

Curves corresponding to smaller (9.525 mm) and slightly larger (15.875 mm) diameters also approximate the measurements but deviate more noticeably at shallow and deep covers, respectively. For diameters exceeding 19 mm, the predicted amplitudes become significantly higher than the measured values, reflecting stronger theoretical reflections that were not observed in the tests. This divergence supports the error analysis in Table 4, where *E_a_* increased substantially for diameters larger than the actual reinforcement. The close grouping of measured data around the 12.7 mm and 9.525 mm curves further indicates that the amplitude-based method can effectively distinguish reinforcement diameters within this range. The quantitative comparison of these amplitude-depth relationships is summarized in Table 5, which presents the mean, standard deviation, and median of the normalized amplitude prediction errors for each candidate diameter.

Table 5 summarizes the normalized amplitude prediction errors (*E_a_*) computed for different reinforcement diameters when compared with measured data from beams reinforced with 12.7 mm bars. The lowest mean and median errors of 4.71% and 3.20%, respectively, were obtained for the 12.7 mm curve, indicating that this diameter provides the closest match to the measured amplitude-depth trend. Among the 83 analyzed locations, 58% of cases showed the minimum error corresponding to the 12.7 mm curve, while 28.4% corresponded to the 9.525 mm curve and smaller proportions to the larger diameters. These proportions suggest that the majority of reflections are best represented by the 12.7 mm response, consistent with the actual reinforcement used in the specimens.

For the remaining cases, slightly higher errors (mean 7–10%) were observed for the 9.525 mm and 15.875 mm curves, and substantially larger errors for diameters above 19 mm, reaching more than 20%. The systematic increase in *E_a_* with assumed diameter reflects the mismatch between the measured amplitude attenuation and the modeled response for incorrect bar diameters. Overall, the results indicate that the proposed amplitude-depth comparison method effectively differentiates between reinforcement diameters based on quantitative error analysis, with the lowest *E_a_* values and highest occurrence frequency corresponding to the true bar diameter.

## 6. Conclusions

This study developed a methodology for evaluating reinforced concrete structures using ground-penetrating radar (GPR) by establishing a normalized amplitude-diameter-depth (NADD) relationship derived from an exponential attenuation model. The NADD chart was constructed from GPR measurements of a concrete slab containing reinforcement bars with known diameters and depths and then applied to beam data to identify reinforcement bar diameters.

The comparison between measured and predicted normalized amplitudes showed a mean prediction error (*E_a_*) of approximately 4.7% for the 12.7 mm bars, corresponding to the actual reinforcement used in the beams. Errors for other assumed diameters increased progressively, confirming that the model effectively distinguishes diameter-dependent amplitude behavior within the tested range.

The proposed NADD-based approach provides a quantitative and repeatable framework for interpreting GPR amplitude-depth data in reinforced concrete members. Future work will extend this analysis to concrete with varied material compositions and field conditions to further evaluate the method’s robustness and applicability.

## Figures and Tables

**Table 1:** Parameters of the GPR device.

Description	Unit	Value
Level of the antenna	m	0.08
Scan depth	m	0.8
Scan spacing in a scan line	mm	Varies
Antenna frequency	GHz	1.6
Samples per scan	-	2048
Scans per meter	-	300

**Table 2:** Values of *α*(*D*) and *A*_1_(*D*).

Reinforcement Bar Diameter (mm)	*α*(*D*)	*A*_1_(*D*)	*R* ^2^
9.525	0.030	4.6	0.987
12.7	0.031	5.3	0.985
15.785	0.032	6.0	0.983
19.05	0.033	7.0	0.983
22.225	0.034	7.7	0.984
25.4	0.043	15.4	0.987

**Table 3:** Parameters of resolution.

Beam	Samples per Scan	Scan per Meter
1 to 3	2048	886

**Table 4:** Input Analysis Parameters.

Beam	Time-Zero (ns)	EM Wave Velocity (mm/ns)
1 to 3	1.30	125

**Table 5:** Normalized amplitude prediction error (*Eₐ*) by reinforcement bar diameter.

Diameter (mm)	Mean (%)	SD (%)	Median (%)	n
9.525	7.28	3.77	7.18	25
12.7	4.71	4.52	3.20	51
15.875	10.21	6.27	9.10	9
19.05	21.22	8.44	20.84	3
22.225	26.33	9.49	25.96	0
25.4	54.15	20.59	56.44	0

## Data Availability

The data presented in this study are available on request from the corresponding author due to restrictions from the funding agency.
